# Cardiac function in 6-year-old children born extremely preterm and associations to prolonged patent ductus arteriosus shunting

**DOI:** 10.1038/s41598-025-34302-x

**Published:** 2026-01-09

**Authors:** Jonna Karlén, Lilly-Ann Mohlkert, Anna Gudmundsdottir, Håkan Eliasson, Anna-Karin Edstedt Bonamy, Cecilia Pegelow Halvorsen

**Affiliations:** 1 Department of Clinical Science and Education, Stockholm South General Hospital (Södersjukhuset), Karolinska Institutet, Stockholm, Sweden; 2 Department of Neonatology at Sachs’ Children and Youth Hospital, Stockholm South General Hospital (Södersjukhuset), Stockholm, Sweden; 3 Pediatric Cardiology Department at Sachs’ Children and Youth Hospital, Stockholm South General Hospital (Södersjukhuset), Stockholm, Sweden; 4 Department of Women’s and Children’s Health, Karolinska Institutet, Stockholm, Sweden; 5 Department of Neonatology, Landspitali University Hospital, Reykjavik, Iceland; 6 Pediatric Cardiology Department, Astrid Lindgren Children’s Hospital, Karolinska University Hospital, Stockholm, Sweden; 7 Clinical Epidemiology Division, Department of Medicine Solna, Karolinska Institutet, Stockholm, Sweden

**Keywords:** Cardiac outcome, Echocardiography, Extremely preterm, Longitudinal study, Patent ductus arteriosus, Neonatology, Paediatric research, Preterm birth, Echocardiography, Cardiology

## Abstract

Follow-up studies of extremely preterm born (EPT) individuals have shown reduced cardiac dimensions and altered biventricular function compared with term born controls. A hemodynamically significant patent ductus arteriosus (hsPDA) increases cardiac load during the neonatal period but potential long-term risks associated with prolonged hsPDA shunting are still largely unknown. We aimed to describe cardiac dimensions and functions in EPT children at 6.5 years of age and to investigate if a prolonged neonatal hsPDA shunt would have a negative cardiac impact. In this follow-up study, EPT children underwent repeated neonatal echocardiograms to evaluate PDA shunt duration. At 6.5 years of age, echocardiography was performed for measurements of dimensions and functions and compared with age matched term born controls. Subgroup analyses were made according to neonatal PDA shunt duration. EPT children (*n* = 57) had significantly smaller heart chambers, a more globe-shaped left ventricle, and signs of altered biventricular systolic function compared with age matched term born controls (*n* = 63). Children exposed to a hsPDA for > 21 days showed no major cardiac alterations compared with children with shorter shunt duration. Our findings support the theory of early cardiac remodeling following EPT birth. Reassuringly, no major cardiac alterations were seen after a prolonged hsPDA shunt.

**Supplementary Information:**

The online version contains supplementary material available at 10.1038/s41598-025-34302-x.

## Introduction

Preterm birth has been associated with cardiac alterations, such as smaller heart chambers^1–12^ and altered right and left ventricular systolic and diastolic functions^1–6,8–11,13,14^. There is also substantial evidence linking prematurity to an increased risk of long-term adverse cardiovascular outcome such as heart failure, ischemic heart disease and systemic hypertension^15–17^.

Following preterm birth, the immature heart must adapt to extra-uterine conditions when exposed to circulatory changes and interruption of normal intrauterine cardiac development. A well-known factor influencing the neonatal hemodynamic load is the presence of a hemodynamically significant patent ductus arteriosus (hsPDA). PDA has been associated with an increased risk of necrotizing enterocolitis (NEC)^18^, intraventricular hemorrhage (IVH) and bronchopulmonary dysplasia (BPD)^19^ in extremely preterm (EPT) infants (born before gestational age (GA) 28 weeks). There is lack of consensus among neonatologists regarding the definition of a hsPDA, when screening for PDA should be performed, and if PDA treatment should be initiated^20^. In many centers, PDA treatment strategy has shifted from early targeted treatment (< 72 h) to a more conservative approach^20^ due to the absence of proven benefits of PDA treatment on long-term outcomes^21–23^ and supported by studies showing high spontaneous closure rates^24,25^. As more ductal shunts are left untreated, there is a growing concern regarding possible risks of BPD and pulmonary vascular disease related to a prolonged PDA shunt duration^26,27^. These findings suggest that the hsPDA could be a contributing factor also to cardiac remodeling in preterm born individuals. The potential effects of a prolonged hsPDA on long-term cardiovascular outcomes may therefore be highly relevant but are so far uncharted.

In this prospectively collected, observational longitudinal cohort study, we hypothesized that EPT children would exhibit an altered cardiac structure and function at 6.5 years, compared with age-equivalent term born controls (CTRL), and that exposure to a hsPDA shunt > 21 days would enhance these alterations.

## Materials and methods

### Study cohort

This study was conducted at the Department of Pediatric cardiology at Stockholm South General Hospital in Sweden. The study population was recruited from a prospective cohort of EPT infants admitted to the Neonatal Intensive Care unit at Karolinska University Hospital in Stockholm, Sweden, from February 14^th^, 2012 until July 31^st^, 2014^28^. Initial exclusion criteria were cardiac or other major congenital malformations. All survivors were invited to participate in this follow-up study at 6.5 years of age. From height and weight measurements, body mass index (BMI) was calculated as weight (kg)/height (cm)^2^, and body surface area (BSA) was calculated according to Haycock formula^29^. To measure heart rate, systolic and diastolic blood pressures, an oscillometric device, GE®, DINAMAP, Carescape V10 (GE HealthCare, Germany) was used. Blood pressures were measured on right arm with the child in a half-lying position. The medical history of participants and their parents, including parental smoking and family history of cardiovascular disease was obtained. As reference, we used a control group of 63 healthy children, born term and without congenital heart or lung disease, who had been assessed with echocardiography in our setting for the EXPRESS-CHARM study at 6.5 years of age^3^. The CTRL children were not matched individually to the EPT-born children.

### Neonatal characteristics and determination of PDA status during the neonatal period

Data on neonatal characteristics were extracted from medical charts during the original study^28^. Morbidities during the neonatal period that were accounted for in this study were NEC according to Bell’s criteria^30^, IVH as graded by Papile^31^, severe BPD defined as need of ≥30% oxygen or positive pressure ventilation at 36 weeks postmenstrual age, sepsis defined as clinical symptoms and blood samples indicating an infection and at least one positive blood culture, and retinopathy of prematurity (ROP) defined according to The International Classification of Retinopathy of Prematurity^32^. Small for gestational age (SGA) was defined as a birth weight below -2 standard deviations (SD) according to Swedish reference growth curves^33^. Asthma in CTRL children was defined as use of asthma medication or any episode of respiratory wheeze within 12 months before follow-up as stated by parents, and in EPT children it was stated by caregivers in questionnaire.

All eligible infants had at least one extensive echocardiographic assessment to rule out congenital structural heart defects and subsequent examinations were performed to evaluate the PDA until it was considered closed^28^. All neonatal echocardiograms were re-examined off-line by the neonatal study investigator (AG) and the definition of a hsPDA during the neonatal period was based on a combination of clinical signs and echocardiography data according to local regional hospital guidelines at that time^28^. Any PDA treatment was at the discretion of the neonatologist in charge. For two children who had not closed their PDA before transfer to another hospital, information on later PDA status was, for the follow-up study, and after parental consent, collected from local hospital medical records.

### Definition of neonatal hsPDA shunt duration for the follow-up study

For the follow-up study, a hsPDA shunt during the neonatal period was redefined based on the criteria of a moderate-to-large PDA for the PDA-TOLERATE trial by Clyman et al.^21^; an internal ductus diameter ≥1.5 mm; and at least one of the following: left atrial to aortic root ratio (LA:Ao) ≥ 1.6, ductus maximum flow velocity ≤ 2.5 m/s, left pulmonary artery diastolic flow velocity > 0.2 m/s, and/or reversed diastolic flow in the descending aorta. If the ductal diameter was missing, data on two other variables were considered enough to determine the hemodynamic significance of the duct. If data was only available for one variable, that examination was considered missing and if data was available for two or more variables, but the criteria as mentioned above were not met, the PDA was defined as not hemodynamically significant. To define the shunt duration of the hsPDA, we measured the numbers of days from the first echocardiogram meeting criteria of hsPDA to the first echocardiogram showing no hsPDA. The infants with a hsPDA shunt during the neonatal period were categorized into three groups according to shunt duration: 0–7 days, 8–21 days and > 21 days, respectively.

### Echocardiographic assessments at 6.5-year follow-up

All echocardiographic examinations at 6.5 years of age were performed by an experienced cardiac sonographer (LAM) with a multifrequency 8–3 MHz vector wide view array transducer for Acuson SC2000 (Siemens® Medical Solutions, USA). Off‐line image analyses (Syngo Dynamics, Siemens USA) of dimensions and functional variables for the study group were performed by one operator (JK) and off-line strain analyses were performed by the sonographer (LAM). Interobserver reproducibility analyses were performed for selected echocardiographic variables and expressed as coefficients of variation.

### Cardiac dimensions

**Fig. 1:**
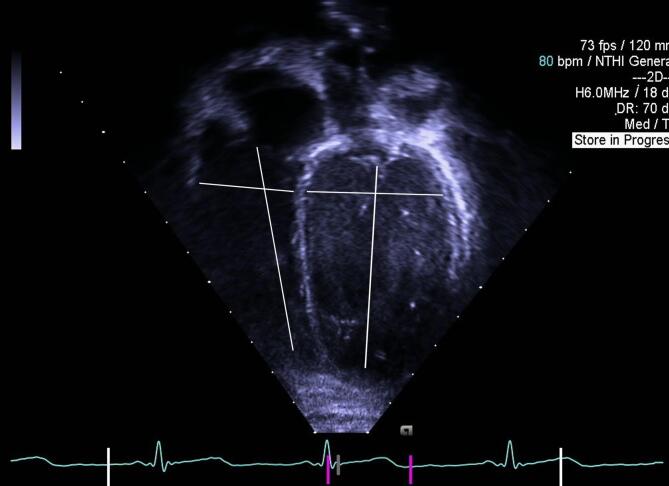
Measurements of longitudinal length and transverse width in end-diastole of left ventricle (LV) and right ventricle (RV) ffrom an apical 4-chamber view (Ap4C).

Assessments of cardiac dimensions and volumes were based on standardized views for pediatric echocardiograms according to guidelines of the American Society of Echocardiography^34,35^. Longitudinal length and transverse width in end-systole of the left atrium (LA) and the right atrium (RA), and in end-diastole of left ventricle (LV) and right ventricle (RV) were measured from an apical 4-chamber view (Ap4C) (Fig. 1). Aortic valve (AoV) in parasternal long-axis view (PLAX) and pulmonary valve (PV) annuli in parasternal short-axis view were measured at hinge point of the valves. Sphericity index (SI) was calculated as length/width for each ventricle. LV posterior wall, interventricular septum (IVS) and aorta were measured in diastole and LA in systole using M-mode in PLAX, and LA to aortic root ratio (LA:Ao) was calculated. The LV relative wall thickness was calculated as (IVS + LV posterior wall)/LV width.

### Systolic and diastolic functions

Left and right systolic global function was measured longitudinally by mitral annular plane systolic excursion (MAPSE) and tricuspid annular plane systolic excursion (TAPSE), using M-mode in Ap4C. Left and right global systolic myocardial velocity (s’) were measured in the annular septal and lateral walls in Ap4C using tissue Doppler imaging (TDI). Left ventricular end-diastolic diameter (LVED) and end-systolic diameter (LVES), were measured using M-mode in PLAX and, shortening fraction was calculated (LVED – LVES)/ LVED. Using TDI, septal and lateral systolic ejection time (ET), isovolumic contraction time (ivct’) and isovolumic relaxation time (ivrt’) were measured and myocardial performance index was calculated using the formula (ivct’ + ivrt’)/ET.

RV stroke volume (SV) was calculated with velocity time integral for right ventricular outflow tract (RVOT_vti_) estimated by pulsed wave Doppler and PV annulus diameter using the formula (π × PV annulus^2^ /4 × RVOT_vti_) and LV SV was calculated with velocity time integral for left ventricular outflow tract (LVOT_vti_) and AoV annulus diameter, using the formula (π × AoV annulus^2^ /4 × LVOT_vti_). Cardiac output (CO) was calculated as SV x heart rate/min.

Using two‐dimensional speckle tracking echocardiography, longitudinal myocardial deformation of LV during systole was assessed at 70–90 frames per second using velocity vector imaging software (VVI, v3.0; Siemens Acuson Medical Solutions). The LV endocardial border was manually traced to obtain global longitudinal peak systolic strain percentage. To assess diastolic function, right and left ventricular early (e’) and late (a’) myocardial velocities (cm/s) were measured by TDI, and the e’/a’ ratio was calculated.

### Statistical analyses

All statistical analyses were performed using STATA/IC 15.1 (StataCorp LLC, Collage Station, TX, USA). Shapiro-Wilks test, visual inspection of data distributions of each variable and skewness (between −1 and + 1) were used to test for normality. Results were reported as means and SD for normally distributed variables and as median and interquartile range (25th to 75th percentile; IQR) for non-normally distributed variables. Proportions were presented as numbers and percentages. Due to multiple comparisons and to reduce the risk of mass significance, a *p*-value < 0.01 was considered statistically significant.

To compare children born EPT and term born CTRL, we used Student’s t-test for normally distributed variables, and Mann Whitney U-test or Wilcoxon rank sum test for non-normally distributed variables. For all echocardiographic variables, adjusted mean differences with 95% confidence interval were calculated using multiple linear regression with BSA and sex as independent variables. To test for differences in proportions, Chi-square test or Fisher’s exact test were used. For sub-group analyses within the EPT group, stratified by duration of a hemodynamically significant ductal shunt in days, we used ANOVA for normally distributed data. For non-normally distributed data, Kruskal Wallis test and Dunn’s test for pairwise post-hoc comparisons were used. Within the EPT group, we performed bivariate analyses with GA and SGA respectively as independent variables. We finally stratified for GA comparing children born at GA 23 + 0–25 + 6 weeks with those born at 26 + 0–27 + 6 weeks.

### Ethics Statement

The study was conducted according to the guidelines of the Declaration of Helsinki and approved by the Regional Ethics Review Board in Stockholm with reference numbers 2011/772-31/4 (2011-06-08), amendment 2012/1253-32 (2012-07-12), 2018–556-31/1 (2018-04-27) and 2010/520‐31/2 (2010-05-15), amendment 2011/376‐32 (2011-04-14). Written signed consent was obtained from legal guardians of all subjects involved in the study.

## Results

### Study cohort

**Fig. 2:**
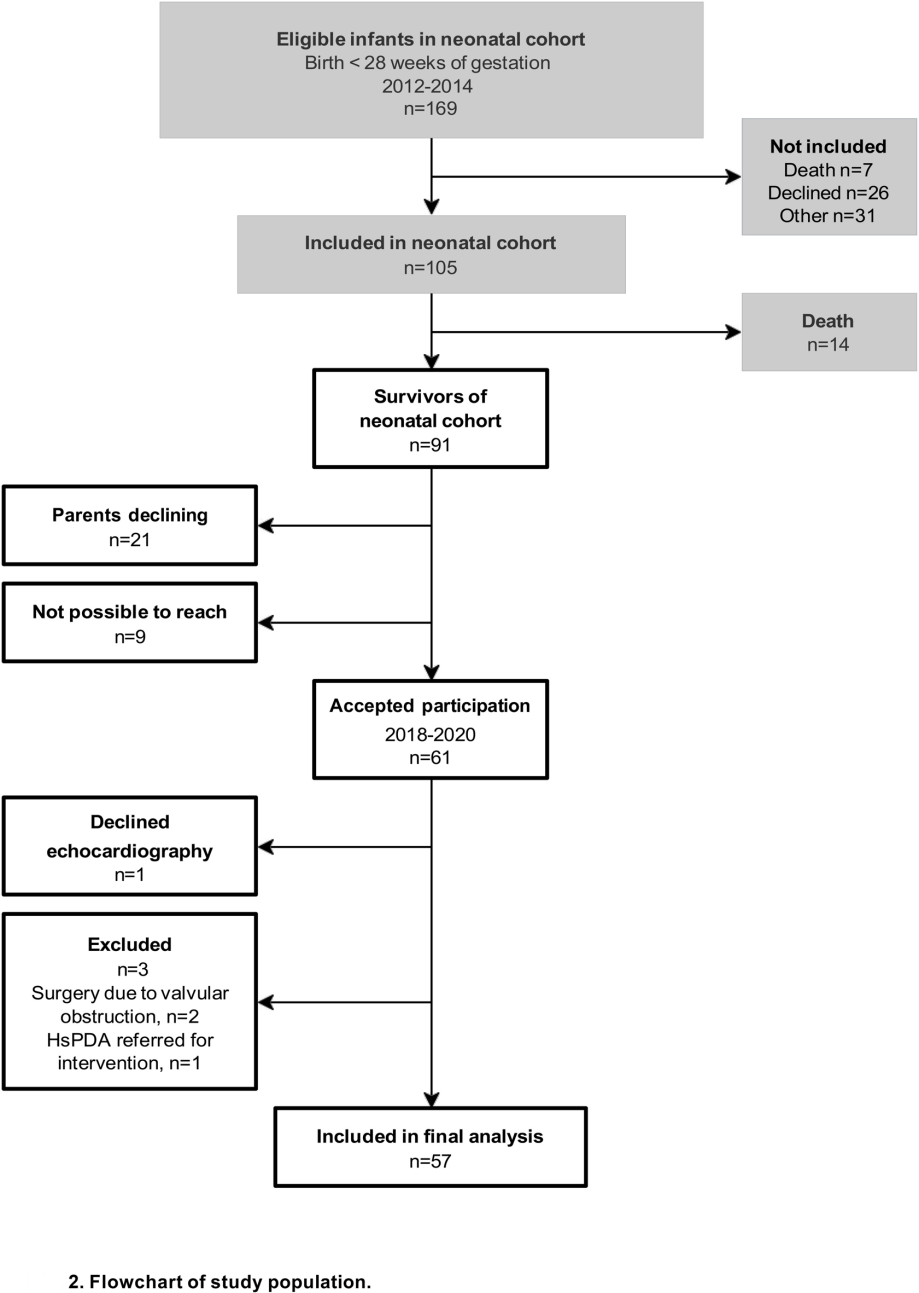
Flow chart of study population.

Of 91 eligible survivors of the neonatal cohort, the legal guardians of 61 (69%) children consented to participation in the follow-up study, and all children were examined at 6.5 years of age (± 3 months) (Fig. 2). We excluded three participants for the final analyses: two had undergone catheter intervention due to pulmonary valvular obstructions and one was diagnosed with a hsPDA at follow-up examination and therefore referred for percutaneous ductal closure. Four participants had trivial mitral valve regurgitations, two had bicuspid aortic valves without stenosis or regurgitation and one had a small ventricular septal defect, none of these were excluded from the study analyses.

### Neonatal and follow-up characteristics

Neonatal and follow-up characteristics are described in Tables 1 and 2. Antenatal steroids were given in all cases of EPT birth. In drop-out analysis, comparing participants (n = 57) versus survivors of the neonatal cohort declining participation (n = 30), we found no significant differences in neonatal characteristics or major neonatal morbidities (see Supplementary Table S1 online). Date for PDA closure was obtained for all included children.

**Table 1:** Neonatal and follow-up characteristics in 6.5-year-old children born extremely preterm (EPT) and controls born at term (CTRL). EPT children stratified by duration with a moderate-to-large hemodynamically significant PDA shunt.

	CTRL n = 63	EPT all children n = 57	*P*-value	EPT children with PDA shunt 0–7 days n = 20	EPT children with PDA shunt 8–21 days n = 21	EPT children with PDA shunt > 21 days n = 16
Neonatal data						
Gestational age, weeks, mean (SD)	39.9 (1.1)	25.9 (1.2)	< 0.001*	26.3 (1.2)	25.7 (1.3)	25.7 (1.2)
Birthweight, grams, mean (SD)	3569 (537)	803 (187)	< 0.001*	803 (215)	818 (184)	784 (163)
BW SDS, (SD)	0.02 (1.1)	−1.1 (1.3)	< 0.001*	−1.4 (1.4)	−0.8 (1.1)	−1.0 (1.2)
SGA^a^, n (%)	2 (3)	12 (21)	0.003*	5 (25)	3 (14)	4 (25)
Boys/girls, n (%)	37/26 (59/42)	26/31 (46/54)	0.15	10/10 (50/50)	11/10 (52/48)	5/11 (31/69)
6.5-year follow-up						
Age at exam, years, mean (SD)	6.6 (0.1)	6.6 (0.1)	0.91	6.6 (0.1)	6.5 (0.1)	6.6 (0.2)
Weight, kilograms, mean (SD)	24.7 (4.2)	19.9 (3.6)	< 0.001*	19.0 (2.4)	20.8 (5.2)	19.7 (2.0)
Height, centimeters, mean (SD)	122.5 (4.7)	116.6 (5.2)	< 0.001*	115.0 (5.5)	117.4 (5.1)	117.3 (4.8)
BMI, kg/m^2^, mean (SD)	16.4 (2.3)	14.6 (1.8)	< 0.001*	14.4 (1.4)	14.9 (2.5)	14.3 (1.3)
BSA, m^2^, mean (SD)	0.91 (0.09)	0.80 (0.08)	< 0.001*	0.78 (0.06)	0.82 (0.11)	0.80 (0.05)
HR, bpm, mean (SD)	87 (9)^g^	84 (11)	0.15	84 (10)	84 (14)	84 (10)
SBP, mmHg, mean (SD)	99 (7)	97 (8)	0.23	97 (7)	98 (9)	96 (7)
DBP, mmHg, mean (SD)	56 (6)	57 (5)	0.31	58 (7)	56 (5)	55 (3)
Smoking^b^, n (%)	12 (21)	10 (16)	0.49	3 (15)	4 (19)	5 (31)
Smoking during pregnancy^c^, n (%)	8 (14)	7 (11)^g^	0.60	2 (10)	2 (10)	4 (25)
Hypertensive disorder^d^, n (%)	7 (13)^f^	N/A	N/A	3 (16)^g^	3 (15)^g^	1 (7)^g^
Family history of CVD^e^, n (%)	6 (11)^f^	3 (5)	0.30	1 (5)^g^	2 (10)^g^	3 (20)^g^
Asthma, n (%)	16 (28)	2 (3)	< 0.001*	5 (25)	6 (29)	5 (31)

Body mass index (BMI); body surface area (BSA); birth weight standard deviation score (BW SDS); cardiovascular disease (CVD); diastolic blood pressure (DBP); heart rate (HR) interquartile range (IQR); not applicable (N/A); patent ductus arteriosus (PDA); systolic blood pressure (SBP); standard deviation (SD); small for gestational age (SGA).

Significant difference, *P*-value < 0.01.

^a^ SGA defined as a birth weight below −2 standard deviations (SD) according to reference Swedish growth curves. ^b^ Any caregiver smoking before or during pregnancy or during their child’s first year of life. ^c^ Any caregiver. ^d^ Preeclampsia, essential hypertension or Hemolysis, Elevated Liver enzymes, Low Platelets (HELLP). ^e^ A history of myocardial infarction, coronary bypass surgery, hypertension, stroke or hyperlipidemia in any caregiver.

^f^ Data missing on three participants. ^g^ Data missing on one participant.

**Table 2:** Neonatal characteristics in 6.5-year-old children born extremely preterm (EPT) stratified by duration with a moderate-to-large hemodynamically significant PDA shunt.

	EPT all children n = 57	EPT children with PDA shunt 0–7 days n = 20	EPT children with PDA shunt 8–21 days n = 21	EPT children with PDA shunt > 21 days n = 16
Surfactant treatment, n (%)	47 (82)	14 (70)	17 (81)	16 (100)
Mechanical ventilation, n (%)	46 (81)	15 (75)	16 (76)	15 (94)
Mechanical ventilation, days, median (IQR)	7 (2–24)	6 (1–13)	7 (2–25)	12 (4–35)
CPAP, days, median (IQR)	32 (23–43)	34 (24–47)	35 (25–43)	29 (21–36)
Days with respiratory support^a^, median (IQR)	45 (34–56)	42 (33–53)	49 (34–59)	42 (39–56)
Inotropic support, n (%)	20 (35)	3 (15)	11 (52)	6 (38)
Severe BPD^b^, n (%)	6 (12)^g^	4 (22)^h^	2 (10)^i^	0^i^
Sepsis^c^, n (%)	24 (42)	8 (40)	6 (29)	10 (63)
NEC stage IIb or higher^d^, n (%)	11 (19)	3 (15)	2 (10)	6 (38)
IVH^e^ grade ≥ 3, n (%)	6 (11)	2 (10)	2 (10)	2 (13)
ROP^f^ grade ≥ 3, n (%)	9 (16)	4 (20)	2 (10)	3 (19)
PDA treatment, only ibuprofen, n (%)	23 (40)	8 (40)	7 (33)	8 (50)
PDA treatment, only surgery, n (%)	4 (7)	2 (10)	2 (10)	0
PDA treatment, ibuprofen and surgery, n (%)	10 (18)	0	6 (29)	4 (25)
No PDA treatment, n (%)	20 (35)	10 (50)	6 (29)	4 (25)

Bronchopulmonary dysplasia (BPD); continuous positive airway pressure (CPAP); intraventricular hemorrhage (IVH); interquartile range (IQR); not applicable (N/A); necrotising enterocolitis (NEC); patent ductus arteriosus (PDA); retinopathy of prematurity (ROP).

^a^ Mechanical ventilation and/or CPAP. ^b^ Severe BPD defined as need of ≥ 30% oxygen or positive pressure ventilation at 36 weeks postmenstrual age. ^c^ Sepsis defined as clinical symptoms and blood samples indicating an infection and at least one positive blood culture.

^d^ NEC as graded by Bell’s. ^e^ IVH as graded by Papile. ^f^ ROP defined according to The international Classification of Retinopathy of Prematurity. ^g^ Data missing on five participants. ^h^ Data missing on two participants. ^i^ Data missing on one participant.

### Cardiac dimensions and functions at 6.5 years of age

All children had cardiac dimensions and functional variables within normal range^36,37^. Compared with CTRL children, EPT children had significantly smaller biventricular length, RV width, atrial dimensions, AoV and PV annuli, and more globally shaped LV (Table 3). TAPSE and RV s’ of the free wall, MAPSE, LV s’ of lateral and septal walls, LV SV and CO were significantly lower in EPT compared with CTRL group (Table 4). Septal ivrt’ and ivct’ were shorter, mitral e’ and a’ and mitral and septal myocardial performance index were lower in EPT children compared with CTRL (Table 4 and Supplementary Table S2). No other significant difference was seen in systolic or diastolic functional variables.

**Table 3:** Right and left heart dimensions in 6.5-year-old children born extremely preterm (EPT) and controls born at term (CTRL).

	Accepted^a^ EPT/CTRL	EPT n = 57	CTRL n = 63	*P*-value†	Adjusted mean difference^b^ (95% CI)	*P*-value
Right heart						
RV SI	47/62	1.6 (0.2)	1.8 (0.2)	< 0.001*	−0.2 (−0.3;−0.1)	< 0.001*
RV length, mm	48/62	43.2 (3.9)	53.0 (3.9)	< 0.001*	−8.3 (−10.1;−6.6)	< 0.001*
RV width, mm	47/62	26.6 (1.9)	29.4 (2.8)	< 0.001*	−1.9 (−3.0;−0.8)	0.001*
RA length, mm	50/60	29.2 (2.4)	35.8 (3.6)	< 0.001*	−4.5 (−5.7;−3.3)	< 0.001*
RA width, mm	50/60	26.0 (2.5)	32.0 (3.1)	< 0.001*	−4.4 (−5.5;−3.2)	< 0.001*
PV annulus, mm	50/51	16.1 (1.5)	18.3 (1.8)	< 0.001*	−1.2 (−1.8;−0.5)	0.001*
Left heart						
LV SI	51/62	1.3 (0.1)	1.6 (0.1)	< 0.001*	−0.2 (−0.3;−0.2)	< 0.001*
LV length, mm	51/62	46.6 (3.3)	58.0 (3.8)	< 0.001*	−9.0 (−10.5;−7.6)	< 0.001*
LV width, mm	51/62	34.9 (2.3)	36.9 (2.9)	< 0.001*	−0.3 (−1.3;0.7)	0.57
LA length, mm	50/61	28.5 (2.8)	38.6 (5.0)	< 0.001*	−7.7 (−9.4;−6.0)	< 0.001*
LA width, mm	50/61	24.4 (2.2)	29.5 (3.3)	< 0.001*	−3.4 (−4.6;−2.2)	< 0.001*
AoV annulus, mm	56/55	13.6 (0.8)	15.5 (1.0)	< 0.001*	−1.4 (−1.7;−1.0)	< 0.001*
IVS^c^, mm	57/63	5.4 (0.7)	6.1 (0.8)	< 0.001*	−0.2 (−0.6;0.07)	0.12
LVED^c^, mm	57/63	35.7 (2.5)	38.5 (2.4)	< 0.001*	−0.9 (−1.7;−0.04)	0.040
LVES^c^, mm	57/63	23.8 (2.1)	25.0 (2.5)	0.007*	0.1 (−0.8;0.10)	0.79
PW^c^, mm	57/63	5.6 (0.6)	5.7 (0.7)	0.40	0.2 (−0.1;0.4)	0.21
RWT	51/62	0.31 (0.03)	0.32 (0.04)	0.24	−0.001 (−0.02;0.01)	0.86
LA^c^, mm	55/60	24.4 (3.7)	24.5 (3.5)	0.86	1.6 (0.1;3.1)	0.033
Aorta^c^, mm	55/61	18.3 (1.7)	20.3 (2.2)	< 0.001*	−0.8 (−1.6;−0.06)	0.034
LA:Ao ratio	55/60	1.3 (0.2)	1.2 (0.2)	0.005*	0.1 (0.03;0.2)	0.011

Aortic valve (AoV); Body surface area (BSA); Confidence interval (CI); intraventricular septum (IVS); left atrium (LA); left atrium to aortic root (LA:Ao); left ventricle (LV); left ventricle end-diastolic diameter (LVED); left ventricle end-systolic diameter (LVES); pulmonary valve (PV); posterior wall (PW); right atrium (RA); right ventricle (RV); relative wall thickness (RWT); standard deviation (SD); sphericity index (SI).

Data are shown as mean (SD).

Significant difference, *P*-value < 0.01.

^†^ Crude value. ^a^ Accepted for analysis. ^b^ Mean difference adjusted for sex and BSA.^c^ Measured with M-mode.

**Table 4:** Right and left heart systolic functions in 6.5-year-old children born extremely preterm (EPT) and controls born at term (CTRL).

	Accepted^a^ EPT/CTRL	EPT n = 57	CTRL n = 63	P-value†	Adjusted mean difference^b^ (95% CI)	*P*-value
Right heart						
RVOT_vti_, m	56/62	0.12 (0.02)	0.11 (0.02)	< 0.001*	0.01 (0.006;0.02)	< 0.001*
TAPSE, mm	55/58	18.0 (2.2)	20.9 (2.3)	< 0.001*	−2.0 (−3.0;−1.1)	< 0.001*
GLS, %	41/59	−26.4 (3.9)	−23.9 (4.1)	0.003*	−2.2 (−4.3;−0.2)	0.033
SV, ml	49/50	25.3 (5.9)	29.6 (7.1)	0.001*	−0.7 (−3.3;2.0)	0.63
CO, L/min	49/49	2.2 (0.6)	2.6 (0.5)	0.001*	−0.06 (−0.3;0.2)	0.60
TDI s’, cm/s	54/51	9.6 (1.5)	11.4 (1.5)	< 0.001*	−1.9 (−2.5;−1.2)	< 0.001*
Left heart						
LVOT_vti_, m	52/60	0.15 (0.02)	0.14 (0.02)	0.73	0.004 (−0.007;0.02)	0.45
MAPSE, mm	54/57	11.1 (1.6)	13.2 (1.5)	< 0.001*	−2.0 (−2.7;−1.3)	< 0.001*
GLS, %	41/62	−21.7 (3.3)	−20.2 (3.3)	0.027	−1.6 (−3.3;0.1)	0.066
Shortening fraction, %	57/63	0.33 (0.04)	0.35 (0.06)	0.048	−0.02 (−0.04;0.003)	0.090
SV, ml	51/53	20.7 (3.5)	27.3 (5.1)	< 0.001*	−4.4 (−6.4;−2.4)	< 0.001*
CO, L/min	51/52	1.8 (0.4)	2.3 (0.4)	< 0.001*	−0.4 (−0.6;−0.2)	< 0.001*
Septal						
TDI s’, cm/s	56/63	5.9 (0.7)	6.5 (0.7)	< 0.001*	−0.6 (−0.8;−0.3)	< 0.001*
mpi’	55/62	0.37 (0.04)	0.45 (0.06)	< 0.001*	−0.07 (−0.1;−0.05)	< 0.001*
Lateral						
TDI s’, cm/s	54/62	5.6 (0.9)	9.0 (1.4)	< 0.001*	−3.3 (−3.8;−2.8)	< 0.001*
mpi’	53/59	0.38 (0.05)	0.41 (0.07)	< 0.001*	−0.04 (−0.06;−0.009)	0.009*

Body surface area (BSA); confidence interval (CI); cardiac output (CO); global longitudinal strain (GLS) using 2-dimensional speckle tracking echocardiography; left ventricle outflow tract velocity time integral (LVOT_vti_); mitral annular plane systolic excursion (MAPSE); Tissue Doppler imaging-derived myocardial performance index (mpi’); right ventricular outflow tract velocity time integral (RVOT_vti_); standard deviation (SD); stroke volume (SV); tricuspid annular plane systolic excursion (TAPSE); Tissue Doppler imaging-derived annular systolic ejection velocity (TDI s’).

Data are shown as mean (SD).

Significant difference, *P*-value < 0.01.

^†^ Crude value. ^a^ Accepted for analysis. ^b^ Mean difference adjusted for sex and BSA.

### Neonatal characteristics stratified by duration of hsPDA

There were no significant differences in neonatal or follow-up characteristics when stratifying by hsPDA shunt duration (Tables 1 and 2).

### Cardiac dimensions and functions stratified by duration of hsPDA

There were no significant differences in cardiac dimensions related to PDA shunt duration (Table 5). RV SV and CO were significantly smaller and LV septal e’ significantly higher in children with a shunt duration > 21 days compared with children with a shunt duration of 8–21 days (Table 6 and Supplementary Table S3). No other significant differences in cardiac functions were found between shunt groups.

**Table 5:** Right and left heart dimensions in 6.5-year-old in children born extremely preterm (EPT) stratified by duration of a moderate-to-large hemodynamically significant PDA shunt.

	n^a^	EPT children with PDA shunt 0–7 days n = 20	n^a^	EPT children with PDA shunt 8–21 days n = 21	n^a^	EPT children with PDA shunt > 21 days n = 16	*P*-value^b^	*P*-value^c^
Right heart								
RV SI	15	1.7 (0.2)	16	1.6 (0.2)	16	1.6 (0.2)	0.17	0.44
RV length, mm	16	43.0 (4.6)	16	43.7 (4.0)	16	42.8 (3.1)	0.98	0.75
RV width, mm	15	25.7 (2.1)	16	26.7 (2.0)	16	27.3 (1.3)	0.022	0.42
RA length, mm	18	29.3 (2.4)	16	29.0 (2.1)	16	29.5 (2.7)	0.86	0.28
RA width, mm	18	26.0 (2.6)	16	25.8 (2.9)	16	26.2 (1.8)	0.96	0.33
PV annulus, mm	16	15.9 (1.4)	20	16.7 (1.5)	14	15.5 (1.3)	0.23	0.022
Left heart								
LV SI	18	1.4 (0.1)	17	1.3 (0.1)	16	1.3 (0.08)	0.065	0.66
LV length, mm	18	46.6 (3.1)	17	46.7 (3.7)	16	46.5 (3.3)	0.98	0.59
LV width, mm	18	33.9 (2.5)	17	35.3 (1.9)	16	35.7 (2.1)	0.018	0.23
LA length, mm	18	28.4 (2.3)	16	28.3 (2.9)	16	28.9 (3.4)	0.70	0.37
LA width, mm	18	24.0 (1.9)	16	24.4 (2.3)	16	24.8 (2.5)	0.27	0.33
AoV annulus, mm	20	13.4 (0.9)	21	13.8 (0.7)	15	13.6 (0.7)	0.58	0.85
IVS, mm	20	5.3 (0.8)	21	5.6 (0.7)	16	5.3 (0.7)	0.52	0.22
LVED^d^, mm	20	35.1 (2.4)	21	36.2 (2.9)	16	35.8 (2.2)	0.31	0.87
LVES^d^, mm	20	23.5 (1.8)	21	24.2 (2.5)	16	23.7 (1.8)	0.77	0.93
PW^d^, mm	20	5.5 (0.6)	21	5.8 (0.7)	16	5.5 (0.6)	0.99	0.35
RWT	18	0.32 (0.03)	17	0.32 (0.03)	16	0.30 (0.04)	0.11	0.32
LA^d^, mm	19	23.9 (3.7)	21	24.5 (4.6)	15	24.9 (2.4)	0.56	0.54
Aorta^d^, mm	19	18.2 (1.8)	21	18.6 (1.6)	15	17.9 (1.5)	0.39	0.42
LA:Ao ratio	19	1.3 (0.2)	21	1.3 (0.2)	15	1.4 (0.2)	0.37	0.33

Aortic valve (AoV); body surface area (BSA); confidence interval (CI); intraventricular septum (IVS); left atrium (LA); left atrial to aortic root (LA:Ao); left ventricle (LV); left ventricle end-diastolic diameter (LVED); left ventricle end-systolic diameter (LVES); patent ductus arteriosus (PDA); pulmonary valve (PV); posterior wall (PW); right atrium (RA); right ventricle (RV); relative wall thickness (RWT); standard deviation (SD); sphericity index (SI).

Data are shown as mean (SD).

Significant difference, *P*-value < 0.01 adjusted for sex and BSA.

^a^ Accepted for analysis. ^b^ *P*-value comparing PDA shunt > 21 days vs. 0–7 days. ^c^ *P*-value comparing PDA shunt > 21 days vs. 8–21 days.

^d^ Measured with M-mode.

**Table 6:** Right and left heart systolic function in 6.5-year-old children born extremely preterm (EPT) stratified by duration of a moderate-to-large hemodynamically significant PDA shunt.

	n^a^	EPT children with PDA shunt 0–7 days n = 20	n^a^	EPT children with PDA shunt 8–21 days n = 21	n^a^	EPT children with PDA shunt > 21 days n = 16	*P*-value^b^	*P*-value^c^	Adjusted mean difference^d^ (95% CI)
Right heart									
RVOT_vti_, m	19	0.12 (0.02)	21	0.13 (0.02)	16	0.12 (0.01)	0.080	0.026	−0.01 (−0.02;−0.001)
TAPSE, mm	19	17.7 (2.4)	20	17.7 (1.8)	16	19.0 (2.1)	0.098	0.041	1.5 (0.07;2.9)
GLS%	13	−26.3 (4.1)	14	−26.0 (4.5)	14	−26.8 (3.3)	0.80	0.69	−0.6 (−3.6;2.4)
TDI s’, cm/s	17	9.4 (1.9)	21	9.7 (1.4)	16	9.6 (1.2)	0.87	0.98	0.01 (−1.0;1.0)
SV, ml	15	24.6 (5.5)	20	28.2 (6.4)	14	21.9 (2.9)	0.045	< 0.001*	−5.4 (−8.4;−2.4)
CO, L/min	15	2.1 (0.5)	20	2.4 (0.8)	14	1.9 (0.3)	0.13	0.006*	−0.5 (−0.8;−0.1)
Left heart									
LVOT_vti_, m	18	0.14 (0.02)	20	0.15 (0.02)	14	0.14 (0.03)	0.67	0.18	−0.01 (−0.03;0.006)
MAPSE, mm	18	11.2 (1.8)	20	10.7 (1.3)	16	11.6 (1.6)	0.32	0.075	1.0 (−0.1;2.1)
GLS, %	14	−22.3 (3.7)	14	−20.4 (1.9)	13	−22.4 (3.8)	0.96	0.13	−2.0 (−4.6;0.6)
Shortening fraction, (%)	20	0.33 (0.04)	21	0.33 (0.05)	16	0.34 (0.04)	0.53	0.82	0.003 (−0.03;0.03)
SV, ml	18	20.2 (3.7)	20	22.2 (2.5)	13	19.2 (3.9)	0.32	0.030	−2.6 (−4.9;−0.3)
CO, L/min	18	1.7 (0.4)	20	1.9 (0.4)	13	1.6 (0.4)	0.64	0.14	−0.2 (−0.5;0.07)
Septal									
TDI s’, cm/s	19	5.9 (0.6)	21	5.9 (0.7)	16	6.0 (0.7)	0.65	0.26	0.3 (−0.2;0.7)
mpi’	18	0.37 (0.04)	21	0.38 (0.04)	16	0.35 (0.03)	0.12	0.091	−0.02 (−0.05;0.004)
Lateral									
TDI s’, cm/s	17	5.3 (0.7)	21	5.6 (1.1)	16	5.9 (0.8)	0.091	0.24	0.4 (−0.3;1.0)
mpi’	17	0.37 (0.04)	20	0.39 (0.05)	16	0.36 (0.04)	0.19	0.016	−0.04 (−0.07;−0.008)

Body surface area (BSA); confidence interval (CI); cardiac output (CO); global longitudinal strain (GLS) using 2-dimensional speckle tracking echocardiography; left ventricle outflow tract velocity time integral (LVOT_vti_); mitral annular plane systolic excursion (MAPSE); Tissue Doppler imaging-derived myocardial performance index (mpi’); right ventricular outflow tract velocity time integral (RVOT_vti_); standard deviation (SD); stroke volume (SV); tricuspid annular plane systolic excursion (TAPSE); Tissue Doppler imaging-derived annular systolic ejection velocity (TDI s’).

Data are shown as mean (SD).

Significant difference, *P*-value < 0.01, adjusted for sex and BSA.

^a^ Accepted for analysis. ^b^ *P*-value comparing PDA shunt > 21 days vs. 0–7 days. ^c^ *P*-value comparing PDA shunt > 21 days vs. 8–21 days. ^d^ Mean difference comparing PDA shunt > 21 days vs. 8–21 days.

### Perinatal factors and echocardiographic assessments

Neonatal characteristics and cardiac dimensions and functions were analyzed after stratification for GA comparing EPT children born at GA 23 + 0–25 + 6 weeks with those born at 26 + 0–27 + 6 weeks (see Supplementary Table S4–S7 online). Children born before GA 26 weeks had a significantly lower birthweight, spent more days on mechanical ventilation and were more likely to have had ROP grade ≥ 3 (see Supplementary Table S4 online). There were no significant differences in follow-up characteristics or cardiac dimensions or functions (see Supplementary Table S4–S7 online). After adjusting for sex and BSA, there were still no significant differences in cardiac dimensions or functions when comparing EPT children born SGA with those born appropriate for gestational age.

Additionally, the EPT children who underwent neonatal PDA surgery (n = 14) at a median age of 19 days [IQR 15–28] during the neonatal period had a significantly lower mean GA (24.6 weeks ± 1.0 vs. 26.3 weeks ± 1.0, *P* < 0.001) and birthweight (660 g ± 102 vs. 850 g ± 186, *P* = 0.001) than those who only received pharmacological treatment or no treatment at all (n = 43). They also spent more days on mechanical ventilation (32 days [IQR 17–35] vs. 4 days [IQR 0–10], *P* < 0.001) and were more likely to have had inotropic support (14 (100%) vs. 6 (14%), P < 0.001) and had been diagnosed with IVH grade ≥ 3 (5 (36%) vs. 1 (2%), *P* = 0.002) than the rest of the cohort. In multivariate regression analyses with sex and BSA as independent variables, EPT children who underwent PDA surgery (n = 9) had significantly larger LA length (30.9 mm ± 3.6 vs. 28.0 mm ± 2.4, *P* = 0.005) compared with the rest of the cohort (n = 41), with no other significant differences seen in dimensional or functional variables.

### Significance of sex

After adjusting for BSA, EPT boys had significantly shorter RV width than CTRL boys (26.2 mm ± 2.3 vs. 30.1 mm ± 2.9 *P* < 0.001), whereas no significant difference was seen when comparing EPT and CTRL girls (26.9 mm ± 1.5 vs. 28.4 mm ± 2.4, *P* = 0.47 with *P* = 0.006 for interaction).

### Interobserver variability

Interobserver variability expressed as coefficient of variation for echocardiographic assessments of LVED and LVES measured with M-mode, was 1.7–2.2% and for RV and LV global longitudinal peak systolic strain it was 6 and 7% respectively.

## Discussion

In this observational echocardiography-based follow-up study of children born extremely preterm EPT (n = 57), we found smaller ventricular and atrial dimensions, more globe-shaped LV, and signs of altered left and right ventricular systolic functions at 6.5 years of age, compared with an age-matched term born control group (n = 63). EPT children exposed to a prolonged hsPDA shunt > 21 days did not show impaired functional or dimensional cardiac outcome.

Preterm born individuals have been reported to exhibit cardiovascular characteristics^7,11^. Reduced cardiac chamber sizes have been suggested as key findings^1–12^ and demonstrated as early as 36 weeks post menstrual age^38,39^. Our findings of shorter atrial and ventricular lengths in EPT compared with CTRL children are in concordance with this. LV length is an independent predictor of impaired systolic response to physical exercise in preterm born adults, suggesting a link between morphological changes and altered cardiac performance^7^. Therefore, the decrease in LV length compared with a less pronounced decrease in LV width seen in EPT children of our study, is an intriguing finding. In addition, the dimensional changes in the current study indicate more globe-shaped LV, a finding that has been previously observed in preterm born neonates and adolescents^9,12^. Increased LV sphericity could be an adaptive response to reduce wall stress induced by pressure and volume overload, which might have future implications acting as an independent risk factor of heart failure in adults^40^.

RV dysfunction has been demonstrated to be an independent predictor of mortality and morbidity in cardiovascular as well as in chronic respiratory disease^41–43^. According to a meta-analysis by Telles et al., impaired RV systolic function assessed by TAPSE, manifests already during the neonatal period^11^. A few subsequent studies have confirmed those findings in preterm born children and adults and demonstrated reductions in TAPSE and RV s’ in agreement with our findings^8,10,14^. Lower TAPSE values in EPT children of this study may be related to maturational impairment but could also be linked to the concurrent findings of altered LV systolic function, extending results of previous studies^3,9,13^.

We note that LV lateral e’ was significantly lower in EPT children which could be a sign of altered LV compliance and is supported by previous follow-up studies of preterm born individuals^11,13^. However, no other significant differences were found indicating an impaired LV diastolic function. EPT boys had significantly shorter RV width compared with CTRL boys, whereas no such difference was observed among girls. We speculate that this could indicate a gender-related disparity in cardiac remodeling trajectories. However, as a stand-alone finding, it should be interpreted with care.

With a more expectant attitude to neonatal hsPDA treatment^20^, more neonates will likely be exposed to a substantial shunt for a significant period of time^24^, which was also noted in this study. Meanwhile, some studies have shown an increased risk of adverse pulmonary outcomes related to a prolonged ductal shunt^26,27^. There is a paucity of studies evaluating the potential long-term effects of PDA on cardiac outcome. Contrary to our hypothesis, at 6.5 years of age, we found no major alterations in cardiac dimensions or ventricular function in EPT children exposed to the longest hsPDA duration.

Other studies support the notion of preterm birth having significant impact on RV remodeling and function ^1,11,44^. Early RV adaptive changes have been shown in EPT infants with severe PDA, with preoperative signs of impaired ventriculo-arterial coupling, increased RV volume load, and reduced RV free wall strain, followed by near-complete recovery within 24–48 h post-ligation^45,46^. In this cohort, EPT children with a hsPDA shunt duration > 21 days showed subtle signs of altered RV systolic function with increased TAPSE values in combination with reduced RV SV and CO but without convincing findings of altered diastolic function. Although the differences only remained statistically significant for RV SV and CO after adjustments for multiple comparisons, our minor findings are in line with this notion. Reduced stroke work has been ascribed to volumetric limitations, and an exaggerated RV contractile response has been suggested as an adaptive response to reduced volumetric reserve^6^, and as a compensatory mechanism to maintain RV to pulmonary vascular coupling^5^.

Interestingly, EPT children exposed to a hsPDA for 8–21 days exhibited the largest RV volumes. This group were less likely to have developed ROP, NEC, sepsis, or severe BPD compared with the other groups and had a slightly higher birth weight and somewhat larger BSA. We speculate that these children may have been overall healthier, potentially contributing to more favourable long-term RV volumes. Moreover, a relatively larger proportion of this group underwent surgical PDA closure, and we cannot exclude the possibility that these individuals exhibited characteristics that we were unable to account for in our analyses.

EPT children exposed to a hsPDA > 21 days in our cohort displayed a slightly wider RV. Since this difference was not statistically significant, it should be interpreted with care, but together with the concurrent finding in LV width, it could indicate a different ventricular remodeling trajectory following exposure to a prolonged neonatal hsPDA shunt. RV hypercontractility may be related to RV to pulmonary vascular coupling rather than being an adaptive response to decreased ventricular volumes. There are epidemiological studies indicating that individuals born preterm are at later risk of pulmonary hypertension in childhood and as adults^47,48^. Although, we did not find any echocardiographic signs of pulmonary hypertension, e.g. interventricular septal deviation, hypertensive Doppler profiles in pulmonary arteries or high velocity of tricuspid regurgitation, we cannot exclude the possibility of increased pulmonary vascular resistance undetectable by echocardiography or asymptomatic pulmonary hypertension.

EPT children with a hsPDA shunt > 21 days displayed a discrete but non-significant increase in left atrial dimensions and LV width compared with the other two groups. Increased volumes and pressure following postnatal transition^6^, with an additive effect of a prolonged hsPDA shunt during the neonatal period could result in a long-lasting remodeling of left heart chambers. Aligned with this reasoning, EPT children who underwent neonatal PDA surgery had significantly larger LA compared with those only treated pharmacologically or not treated at all. We speculate that these findings could be of future clinical importance as atrial remodeling has been reported to be an important risk factor of later heart failure^6,49^.

Finally, it is plausible that an increased pressure and volume load due to a prolonged hsPDA duration during the neonatal period have a persistent impact on cardiac remodeling, with RV being more vulnerable than LV to permanent alterations. Furthermore, RV structure and function may be more susceptible to continuous insults due to the interaction with pulmonary vasculature and respiratory dysfunction beyond the neonatal period. We believe that larger studies, also including assessments of pulmonary vasculature, are needed before it can be fully established that leaving hsPDA untreated is safe with regard to the development of pulmonary hypertension or other cardiovascular long-term effects.

A strength with this study was its population-based design and the prospectively collected longitudinal echocardiographic data. Moreover, we were able to use consecutive neonatal data on hemodynamical significant echocardiographic variables and time points for ductal closure. The neonatal echocardiographic examinations were performed by different neonatologists, but in order to reduce the risk of information bias, all exams were reviewed off-line by one neonatologist. All echocardiographic examinations at follow-up were performed by the same sonographer, and all off-line measurements were carried out by one and the same neonatologist.

Limitations of the study include the risk of selection bias related to recruitment and drop-outs. Residual confounding must be considered as we lack knowledge about possible characteristics in women delivering preterm compared with women delivering at term. The sample size is relatively small and further limited by the incompleteness or suboptimal quality of some image analyses. This prevents subgroup analyses of some maternal and perinatal factors that could have an impact on cardiovascular remodeling (e.g., preeclampsia, parental smoking and pulmonary morbidity). Furthermore, we did not perform invasive measurements of pulmonary vascular resistance or pulmonary hypertension for this study and could therefore not speculate on a possible effect of altered pulmonary vasculature on our findings. However, from the echocardiographic measurements, there was no such indication.

MAPSE was chosen as a measure of LV systolic function as it has been suggested to be a dependable, robust and easy parameter to obtain compared to ejection fraction (EF)^50^. We recognize that EF by Simpson’s biplane method is a volumetric method to measure LV EF, whereas MAPSE is limited to measuring longitudinal LV function. However, we also measured GLS which is a well-established method to detect subtle changes in LV function. M-mode derived LV function calculations have well-known limitations, and any abnormality in form and or shape as well as abnormal septal movements will significantly reduce the utility of shortening fraction as the calculations are derived from linear measurements and assume a regularly shaped LV.

Due to small sample sizes of hsPDA shunt groups, the ability to draw firm conclusions from the results is restricted. At the same time, differences between groups may not have been detected which may limit conclusions about statistically insignificant differences introducing a risk of type II error. Moreover, to decrease the risk of type I errors following multiple comparisons, we decreased the significance level. Our definition of neonatal hsPDA duration was based on retrospective echocardiographic data using a dichotomous definition (moderate-to-large versus no or mild hemodynamical significance), making it difficult to define the actual magnitude of the shunt. As the PDA is dynamic, it could be non-significant at one point and due to external factors, significant at another point. Hence, our definition of shunt duration must be considered an estimate. Treatment strategy has shifted toward a more conservative approach in our center, and more neonates were subject to surgical closure at the time of the original study than would be today, which may limit generalizability. Infants who underwent PDA surgery might also have had different characteristics as they, at that time, did not only meet the criteria for surgery but were also considered stable enough to undergo the procedure.

A strength of this study is that half of the EPT children were born before GA 26 weeks which makes it relevant when evaluating PDA related outcomes, as this group might be more susceptible to cardiac remodeling. However, all but one of the children who underwent PDA surgery belonged to this group which could limit our conclusions regarding the effect of a prolonged hsPDA on cardiac alterations in the tiniest preterm born children and, may also impact our comparisons of cardiac dimensions and functions related to GA.

## Conclusions

In conclusion, 6.5-year-old children born extremely preterm exhibited smaller heart chambers, a more globe shaped LV and signs of altered biventricular systolic function compared with term born controls. This is the first study to investigate cardiac outcome in children related to the duration of a hsPDA in the neonatal period. Infants with a prolonged hsPDA duration showed no significant differences in cardiac function or chamber dimensions at age 6. However, the detected left ventricular morphological changes and alterations in right ventricular function indicate remodeling of later importance, emphasizing continued follow-up from late childhood into adolescence to confirm or reject the presence of long-term adverse effects. We agree with previous suggestions on cardiovascular follow-up after preterm birth, and more specifically, we recommend policlinic echocardiographic controls after discharge from neonatal care until PDA is considered restrictive or closed.

## Supplementary Information

Below is the link to the electronic supplementary material.

Supplementary Material 1

## Data Availability

Due to the Swedish law that requires users of the data to obtain permission from the Swedish Ethical Review Authority, the datasets generated and analyzed for the current study are not publicly available.

## Abbreviations

a’: Late myocardial diastolic velocity
AoV: Aortic valve
Ap4C: Apical 4-chamber view
BMI: Body mass index
BPD: Bronchopulmonary dysplasia
BSA: Body surface area
CO: Cardiac output
CTRL: Controls
e’: Early myocardial diastolic velocity
EF: Ejection fraction
EPT: Extremely preterm
ET: Ejection time
GA: Gestational age
hsPDA: Hemodynamically significant patent ductus arteriosus
ivct’: Isovolumic contraction time
IVH: Intraventricular hemorrhage
ivrt’: Isovolumic relaxation time
IVS: Interventricular septum
LA: Left atrium
LA:Ao: Left atrium to aortic root ratio
LV: Left ventricle
LVED: Left ventricle end-diastolic diameter
LVES: Left ventricle end-systolic diameter
LVOT_vti_: Velocity time integral for left ventricular outflow tract
MAPSE: Mitral annular plane systolic excursion
NEC: Necrotizing enterocolitis
PDA: Patent ductus arteriosus
PLAX: Parasternal long-axis view
PV: Pulmonary valve
ROP: Retinopathy of prematurity
RV: Right ventricle
RVOT_vti_: Velocity time integral for right ventricular outflow tract
s’: Systolic myocardial velocity
SGA: Small for gestational age
SV: Stroke volume
TDI: Tissue doppler imaging
