# Supplementary material for: Cardiac function in 6-year-old children born extremely preterm and associations to prolonged patent ductus arteriosus shunting
Source: Sci Rep. 2026 Jan 9;16:1291. doi: 10.1038/s41598-025-34302-x (PMC12791129; doi:10.1038/s41598-025-34302-x)
Supplement: Supplementary file 1 — Supplementary Material 1 [file 41598_2025_34302_MOESM1_ESM.docx]

**Cardiac function in 6-year-old children born extremely preterm and associations to prolonged patent ductus arteriosus shunting**

Jonna Karlén^1,2^*, Lilly-Ann Mohlkert^1,3^, Anna Gudmundsdottir^4,5^, Håkan Eliasson^4,6^, Anna-Karin Edstedt Bonamy^7^, and Cecilia Pegelow Halvorsen^1,2,3^

**Affiliations**

^1^ Department of Clinical Science and Education, Stockholm South General Hospital (Södersjukhuset), Karolinska Institutet, Stockholm, Sweden.

^2^ Department of Neonatology at Sachs’ Children and Youth Hospital, Stockholm South General Hospital (Södersjukhuset), Stockholm, Sweden.

^3^ Pediatric Cardiology Department at Sachs’ Children and Youth Hospital, Stockholm South General Hospital (Södersjukhuset), Stockholm, Sweden

^4^ Department of Women’s and Children’s Health, Karolinska Institutet, Stockholm, Sweden.

^5^ Department of Neonatology, Landspitali University Hospital, Reykjavik, Iceland.

^6^ Pediatric Cardiology Department, Astrid Lindgren Children’s Hospital, Karolinska University Hospital, Stockholm, Sweden.

^7^ Clinical Epidemiology Division, Department of Medicine Solna, Karolinska Institutet, Stockholm, Sweden.

*E-mail: jonna.karlen@ki.se

**Supplementary Table S1. Neonatal characteristics in 6.5-year-old children born extremely preterm (EPT) participating in follow-up study compared with non-participants (only survivors).**

|  | **Study cohort**  **n=57** | **Non-participants**  **n=30** | **P-value** |
| --- | --- | --- | --- |
| Gestational age, weeks, mean (SD) | 25.9 (1.2) | 26.1 (1.2) | 0.40 |
| Birth weight, gram, mean (SD) | 803 (187) | 816 (159) | 0.74 |
| BW SDS, mean (SD) | -1.1 (1.3) | -1.2 (1.1) | 0.63 |
| Boys/girls, n (%) | 26/31 (46/54) | 16/14 (47/53) | 0.49 |
| SGA^a^, n (%) | 12 (21) | 9 (30) | 0.35 |
| Surfactant treatment, n (%) | 47 (82) | 23 (77) | 0.52 |
| Mechanical ventilation, n (%) | 46 (81) | 21 (70) | 0.26 |
| Mechanical ventilation, days, median (IQR) | 7 (2-24) | 6 (0-18) | 0.81 |
| CPAP, days, median (IQR) | 32 (23-43) | 34 (22-42) | 0.64 |
| Days in total with respiratory support^b^, median (IQR) | 45 (34-56) | 39 (27-57) | 0.29 |
| Inotropic support, n (%) | 20 (35) | 14 (47) | 0.29 |
| Sepsis^c^, n (%) | 24 (42) | 15 (50) | 0.48 |
| NEC stage IIb or higher^d^, n (%) | 11 (19) | 1 (3) | 0.051 |
| IVH^e^ grade ≥ 3, n (%) | 6 (11) | 5 (17) | 0.41 |
| Severe BPD^f^, n (%) | 6 (12)^h^ | 6 (21)^i^ | 0.24 |
| ROP^g^ grade ≥ 3, n (%) | 9 (16) | 8 (28)^j^ | 0.19 |
| PDA treatment, only ibuprofen, n (%) | 23 (40) | 9 (30) | 0.34 |
| PDA treatment, only surgery, n (%) | 4 (7) | 2 (7) | 1.0 |
| PDA treatment, ibuprofen and surgery, n (%) | 10 (18) | 6 (20) | 0.78 |
| No PDA treatment, n (%) | 20 (35) | 13 (43) | 0.45 |

Birth weight standard deviation score (BW SDS); bronchopulmonary dysplasia (BPD**)**; continuous positive airway pressure (CPAP); intraventricular hemorrhage (IVH); interquartile range (IQR); necrotizing enterocolitis (NEC); patent ductus arteriosus (PDA); retinopathy of prematurity (ROP); standard deviation (SD); small for gestational age (SGA).

Significant difference, P-value < 0.01.

^a^ SGA defined as a birth weight below -2 standard deviations (SD) according to reference Swedish growth curves. ^b^ Mechanical ventilation and/or CPAP. ^c^ Sepsis defined as clinical symptoms together with blood samples indicating an infection and at least one positive blood culture. ^d^ NEC as graded by Bell’s. ^e^ IVH as graded by Papile. ^f^ Severe BPD defined as need of ≥ 30% oxygen or positive pressure ventilation at 36 weeks postmenstrual age. ^g^ ROP defined according to The international Classification of Retinopathy of Prematurity. ^h^ Data missing on five participants. ^i^ Data missing on two non-participants. ^j^ Data missing on one non-participant.

**Supplementary Table S2. Right and left heart diastolic functions in 6.5-year-old children born extremely preterm (EPT) and controls born at term (CTRL).**

|  | **Accepted^a^**  **EPT/CTRL** | **EPT**  **n=57** | **CTRL**  **n=63** | **P-value**† | **Adjusted mean difference^b^**  **(95% CI)** | **P-value** |
| --- | --- | --- | --- | --- | --- | --- |
| **Right heart** |  |  |  |  |  |  |
| TDI e’, cm/s | 54/60 | 13.5 (1.9) | 14.1 (1.8) | 0.091 | -0.7 (-1.5;0.1) | 0.086 |
| TDI a’, cm/s | 54/60 | 6.2 (1.8) | 6.9 (1.7) | 0.050 | -0.5 (-1.3;0.3) | 0.19 |
| e’/a’ | 54/60 | 2.3 (0.7) | 2.2 (0.7) | 0.28 | 0.04 (-0.3;0.4) | 0.81 |
| **Left heart** |  |  |  |  |  |  |
| **Septal** |  |  |  |  |  |  |
| TDI e’, cm/s | 56/63 | 11.4 (1.4) | 12.1 (1.2) | 0.003* | -0.5 (-1.2;-0.03) | 0.040 |
| TDI a’, cm/s | 56/63 | 3.9 (0.9) | 4.3 (0.8) | 0.007* | -0.4 (-0.7;-0.02) | 0.061 |
| e’/a’ | 56/63 | 3.1 (0.9) | 2.9 (0.7) | 0.19 | 0.2 (-0.2;0.5) | 0.35 |
| ivrt’, ms | 55/63 | 47.6 (6.3) | 53.6 (6.7) | <0.001* | -3.9 (-6.6; -1.1) | 0.006* |
| ivct’, ms | 56/63 | 51.5 (7.0) | 64.3 (12.9) | <0.001* | -13.0 (-17.8;-8.3) | <0.001* |
| ET, ms | 55/62 | 270 (16) | 264 (14) | 0.026 | 6.9 (0.2;13.5) | 0.043 |
| **Lateral** |  |  |  |  |  |  |
| TDI e’, cm/s | 54/62 | 15.5 (2.5) | 17.9 (2.6) | <0.001* | -2.4 (-3.5;-1.2) | <0.001* |
| TDI a’, cm/s | 54/62 | 4.2 (1.0) | 5.2 (1.1) | <0.001* | -1.1 (-1.6;-0.6) | <0.001* |
| e’/a’ | 54/62 | 3.8 (0.9) | 3.6 (0.9) | 0.20 | 0.3 (-0.05;0.7) | 0.089 |
| ivrt’, ms | 53/61 | 49.5 (7.3) | 52.6 (8.1) | 0.035 | -2.7 (-6.3;0.8) | 0.13 |
| ivct’, ms | 53/61 | 56.0 (9.2) | 62.3 (12.7) | 0.003* | -5.8 (-10.9;-0.6) | 0.029 |
| ET, ms | 53/59 | 282 (18) | 279 (19) | 0.39 | 4.7 (-4.0;13.4) | 0.29 |

Body surface area (BSA); confidence interval (CI); ejection time (ET); isovolumic contraction time (ivct’); isovolumic relaxation time (ivrt’); standard deviation (SD); Tissue Doppler imaging-derived atrial (late) diastolic velocity (TDI a’); Tissue Doppler imaging-derived early diastolic velocity (TDI e’).

Data are shown as mean (SD).

Significant difference, P-value < 0.01.

† Crude value. ^a^ Accepted for analysis. ^b^ Mean difference adjusted for sex and BSA.

**Supplementary Table S3. Right and left heart diastolic function in 6.5-year-old children born extremely preterm (EPT) stratified by duration of a moderate-to-large hemodynamically significant PDA shunt.**

|  | **n^a^** | **EPT**  **children with**  **PDA shunt**  **0-7 days**  **n=20** | **n^a^** | **EPT children with**  **PDA shunt**  **8-21 days**  **n=21** | **n^a^** | **EPT children with PDA shunt**  **>21 days**  **n=16** | **P-value^b^** | **P-value^c^** | **Adjusted mean difference^d^**  **(95% CI)** |
| --- | --- | --- | --- | --- | --- | --- | --- | --- | --- |
| **Right heart** |  |  |  |  |  |  |  |  |  |
| TDI e’, cm/s | 17 | 13.6 (1.8) | 21 | 13.6 (2.2) | 16 | 13.2 (1.6) | 0.54 | 0.51 | -0.4 (-1.7;0.9) |
| TDI a’, cm/s | 17 | 6.3 (1.9) | 21 | 6.3 (2.0) | 16 | 6.0 (1.3) | 0.49 | 0.73 | -0.2 (1.4;1.0) |
| e’/a’ | 17 | 2.3 (0.7) | 21 | 2.4 (0.8) | 16 | 2.3 (0.6) | 0.85 | 0.70 | -0.09 (-0.6;0.4) |
| **Left heart** |  |  |  |  |  |  |  |  |  |
| **Septal** |  |  |  |  |  |  |  |  |  |
| TDI e’, cm/s | 19 | 11.3 (1.2) | 21 | 10.9 (1.2) | 16 | 12.3 (1.5) | 0.023 | 0.001* | 1.5 (0.6;2.3) |
| TDI a’, cm/s | 19 | 4.1 (0.9) | 21 | 3.8 (0.9) | 16 | 3.8 (1.0) | 0.46 | 0.93 | 0.03 (-0.6;0.7) |
| e’/a’ | 19 | 2.9 (0.7) | 21 | 3.0 (0.9) | 16 | 3.5 (1.2) | 0.080 | 0.16 | 0.5 (-0.2;1.1) |
| ivrt’, ms | 18 | 47.2 (6.0) | 21 | 48.8 (6.6) | 16 | 46.6 (6.2) | 0.50 | 0.24 | -2.5 (-6.6;1.7) |
| ivct’, ms | 19 | 52.9 (9.0) | 21 | 51.9 (6.0) | 16 | 49.4 (5.4) | 0.21 | 0.35 | -2.2 (-7.1;2.6) |
| ET, ms | 18 | 270 (19) | 21 | 267 (16) | 16 | 273 (11) | 0.67 | 0.46 | 3.9 (-6.6;14.4) |
| **Lateral** |  |  |  |  |  |  |  |  |  |
| TDI e’, cm/s | 17 | 14.7 (2.4) | 21 | 15.2 (2.7) | 16 | 16.6 (2.0) | 0.031 | 0.058 | 1.6 (-0.06;3.2) |
| TDI a’, cm/s | 17 | 4.4 (1.3) | 21 | 4.0 (1.0) | 16 | 4.3 (0.7) | 0.65 | 0.57 | 0.2 (-0.5;0.9) |
| e’/a’ | 17 | 3.5 (0.9) | 21 | 3.9 (1.0) | 16 | 4.0 (0.9) | 0.12 | 0.61 | 0.2 (-0.5;0.8) |
| ivrt’, ms | 17 | 49.0 (6.1) | 20 | 50.4 (8.2) | 16 | 48.9 (7.5) | 0.85 | 0.36 | -2.2 (-7.1;2.7) |
| ivct’, ms | 17 | 58.0 (9.3) | 20 | 58.1 (9.5) | 16 | 51.2 (7.1) | 0.033 | 0.035 | -6.5 (-12.5;-0.5) |
| ET, ms | 17 | 287 (20) | 20 | 278 (20) | 16 | 282 (13) | 0.46 | 0.47 | 4.6 (-8.0;17.2) |

Body surface area (BSA); confidence interval (CI); ejection time (ET); isovolumic contraction time (ivct’); isovolumic relaxation time (ivrt’); standard deviation (SD); tissue Doppler imaging-derived atrial (late) diastolic velocity (TDI a’); tissue Doppler imaging-derived early diastolic velocity (TDI e’).

Significant difference, P-value < 0.01.

^a^ Accepted for analysis. ^b^ P-value comparing PDA shunt > 21 days vs. 0-7 days. ^c^ P-value comparing PDA shunt > 21 days vs. 8-21 days. ^d^ Mean difference comparing PDA shunt > 21 days vs. 8-21 days.

**Supplementary Table S4. Neonatal and follow-up characteristics in 6.5-year-old children born extremely preterm (EPT), stratified by gestational age.**

|  | **All children**  **n=57** | **Gestational age**  **23+0-25+6 weeks**  **n=29** | **Gestational age**  **26+0-27+6 weeks**  **n=28** | **P-value** |
| --- | --- | --- | --- | --- |
| **Neonatal characteristics** |  |  |  |  |
| Gestational age, week, mean (SD) | 25.9 (1.2) | 24.9 (0.8) | 26.9 (0.6) | <0.001* |
| Birthweight, gram, mean (SD) | 803 (187) | 717 (128) | 893 (198) | <0.001* |
| SGA^a^, n (%) | 12 (21) | 3 (10) | 9 (32) | 0.056 |
| BW SDS, mean (SD) | -1.1 (1.3) | -0.8 (1.0) | -1.3 (1.5) | 0.08 |
| Boys/girls, n (%) | 26/31 (46/54) | 13/16 (45/55) | 13/15 (46/54) | 0.90 |
| Curosurf, n (%) | 47 (82) | 27 (93) | 20 (71) | 0.031 |
| Mechanical ventilation, n (%) | 46 (81) | 28 (97) | 18 (64) | 0.002* |
| Mechanical ventilation, days, median (IQR) | 7 (2-24) | 15 (7-31) | 3 (0-8) | 0.006* |
| CPAP, days, median (IQR) | 32 (23-43) | 38 (27-44) | 29 (20-37) | 0.062 |
| Days in total with respiratory support^b^, median (IQR) | 45 (34-56) | 54 (48-62) | 34 (26-42) | <0.001* |
| Inotropic support, n (%) | 20 (35) | 13 (45) | 7 (25) | 0.12 |
| Severe BPD^c^, n (%) | 6 (12)^h^ | 2 (7)^i^ | 4 (15)^j^ | 0.67 |
| Sepsis^d^, n (%) | 24 (42) | 13 (45) | 11 (39) | 0.67 |
| NEC stage IIb or higher^e^, n (%) | 11 (19) | 8 (28) | 3 (11) | 0.18 |
| IVH^f^ grade ≥ 3, n (%) | 6 (11) | 6 (21) | 0 | 0.023 |
| ROP^g^ grade ≥ 3, n (%) | 9 (16) | 9 (31) | 0 | 0.002* |
| PDA treatment, only ibuprofen, n (%) | 23 (40) | 10 (34) | 13 (46) | 0.36 |
| PDA treatment, only surgery, n (%) | 4 (7) | 4 (14) | 0 | 0.11 |
| PDA treatment, ibuprofen and surgery, n (%) | 10 (18) | 9 (31) | 1 (4) | 0.012 |
| No PDA treatment, n (%) | 20 (35) | 6 (21) | 14 (50) | 0.020 |
| **6.5-year follow-up** |  |  |  |  |
| Age at exam, years, mean (SD) | 6.6 (0.1) | 6.6 (0.1) | 6.5 (0.1) | 0.41 |
| Weight, kilograms, mean (SD) | 19.9 (3.6) | 19.8 (3.0) | 19.9 (4.2) | 0.96 |
| Height, centimeter, mean (SD) | 116.6 (5.2) | 116.1 (5.2) | 117.0 (5.3) | 0.53 |
| BMI, kg/m^2^, mean (SD) | 14.6 (1.8) | 14.7 (1.5) | 14.4 (2.0) | 0.62 |
| BSA, m^2^, mean (SD) | 0.80 (0.08) | 0.8 (0.07) | 0.8 (0.1) | 0.92 |
| HR bpm, mean (SD) | 84 (11) | 85 (12) | 83 (11) | 0.53 |
| SBP, mmHg, mean (SD) | 97 (8) | 97 (7) | 97 (9) | 0.76 |
| DBP, mmHg, mean (SD) | 57 (5) | 57 (6) | 57 (5) | 1.0 |

Body mass index (BMI); bronchopulmonary dysplasia (BPD); body surface area (BSA); birth weight standard deviation score (BW SDS); continuous positive airway pressure (CPAP); diastolic blood pressure (DBP); heart rate (HR); intraventricular hemorrhage (IVH); interquartile range (IQR); necrotizing enterocolitis (NEC); patent ductus arteriosus (PDA); retinopathy of prematurity (ROP); systolic blood pressure (SBP); standard deviation (SD); small for gestational age (SGA).

Significant difference, P-value < 0.01.

^a^ SGA defined as a birth weight below -2 standard deviations (SD) according to reference Swedish growth curves. ^b^ Mechanical ventilation and/or CPAP. ^c^ Severe BPD defined as need of ≥ 30% oxygen or positive pressure ventilation at 36 weeks postmenstrual age. ^d^ Sepsis defined as clinical symptoms together with blood samples indicating an infection and at least one positive blood culture. ^e^ NEC as graded by Bell’s. ^f^ IVH as graded by Papile. ^g^ ROP defined according to The international Classification of Retinopathy of Prematurity.

^h^ Data missing on five participants. ^i^ Data missing on four participants. ^j^ Data missing on one participant.

**Supplementary Table S5. Right and left heart dimensions in 6.5-year-old children born extremely preterm (EPT), stratified by gestational age.**

|  | **Accepted^a^**  **GA 23-25 weeks**  **/GA26-27 weeks** | **Gestational age 23+0-25+6 weeks**  **n=29** | **Gestational age 26+0-27+6 weeks**  **n=28** | **P-value**† | **Adjusted mean difference^b^**  **(95% CI)** | **P-value** |
| --- | --- | --- | --- | --- | --- | --- |
| **Right heart** |  |  |  |  |  |  |
| RV SI | 23/24 | 1.63 (0.2) | 1.63 (0.2) | 0.97 | 0.0006 (-0.1;0.1) | 0.99 |
| RV length, mm | 24/24 | 43.2 (4.6) | 43.2 (3.2) | 0.95 | 0.1 (-2.1;2.4) | 0.91 |
| RV width, mm | 23/24 | 26.5 (1.6) | 26.7 (2.1) | 0.78 | -0.1 (-1.2;1.0) | 0.84 |
| RA length, mm | 25/25 | 29.3 (2.2) | 29.2 (2.6) | 0.97 | 0.1 (-1.2;1.4) | 0.83 |
| RA width, mm | 25/25 | 25.3 (2.0) | 26.6 (2.7) | 0.058 | -1.1 (-2.4;0.1) | 0.073 |
| PV annulus, mm | 26/24 | 16.2 (1.5) | 16.0 (1.4) | 0.69 | 0.2 (-0.5;0.9) | 0.59 |
| **Left heart** |  |  |  |  |  |  |
| LV SI | 26/25 | 1.34 (0.1) | 1.33 (0.1) | 0.63 | 0.009 (-0.05;0.07) | 0.75 |
| LV length, mm | 26/25 | 46.4 (3.8) | 46.9 (2.6) | 0.57 | -0.3 (-1.9;1.2) | 0.66 |
| LV width, mm | 26/25 | 34.5 (1.9) | 35.4 (2.6) | 0.19 | -0.6 (-1.7;0.5) | 0.28 |
| LA length, mm | 25/25 | 28.4 (3.3) | 28.6 (2.3) | 0.82 | -0.05 (-1.7;1.6) | 0.95 |
| LA width, mm | 25/25 | 24.2 (1.7) | 24.6 (2.7) | 0.48 | -0.3 (-1.6;0.9) | 0.57 |
| AoV annulus, mm | 28/28 | 13.5 (0.7) | 13.7 (0.9) | 0.29 | -0.2 (-0.6;0.2) | 0.26 |
| IVS^c^, mm | 29/28 | 5.5 (0.8) | 5.4 (0.7) | 0.63 | 0.1 (-0.3;0.5) | 0.59 |
| LVED^c^, mm | 29/28 | 35.3 (2.7) | 36.1 (2.3) | 0.23 | -0.7 (-1.9;0.4) | 0.19 |
| LVES^c^, mm | 29/28 | 23.7 (2.0) | 23.9 (2.1) | 0.73 | -0.2 (-1.2;0.8) | 0.76 |
| PW^c^, mm | 29/28 | 5.6 (0.7) | 5.6 (0.6) | 0.82 | -0.03 (-0.4;0.3) | 0.85 |
| RWT | 26/25 | 0.32 (0.04) | 0.31 (0.03) | 0.20 | 0.01(-0.005;0.03) | 0.14 |
| LA^c^, mm | 29/26 | 24.0 (4.1) | 24.8 (3.3) | 0.39 | -0.8 (-2.8;1.1) | 0.40 |
| Aorta^c^, mm | 29/26 | 18.1 (2.0) | 18.5 (1.3) | 0.49 | -0.3 (-1.0;0.5) | 0.45 |
| LA:Ao ratio | 29/26 | 1.3 (0.3) | 1.3 (0.2) | 0.83 | -0.01 (-0.1;0.1) | 0.83 |

Aortic valve (AoV); body surface area (BSA); confidence interval (CI); interventricular septum (IVS); left atrium (LA); left atrial to aortic root (LA:Ao); left ventricle (LV); left ventricle end-diastolic diameter (LVED); left ventricle end-systolic diameter (LVES); patent ductus arteriosus (PDA); pulmonary valve (PV); posterior wall (PW); right atrium (RA); right ventricle (RV); standard deviation (SD); sphericity index (SI).

Data are shown as mean (SD).

Significant difference, P-value < 0.01.

† Crude value. ^a^ Accepted for analysis. ^b^ Mean difference adjusted for sex and BSA. ^c^ Measured with M-mode.

**Supplementary Table S6. Right and left heart systolic function in 6.5-year-old children born extremely preterm (EPT), stratified by gestational age.**

|  | **Accepted^a^**  **GA 23-25 weeks**  **/GA26-27 weeks** | **Gestational age 23+0-25+6 weeks**  **n=29** | **Gestational age 26+0-27+6 weeks**  **n=28** | **P-value**† | **Adjusted mean difference^b^**  **(95% CI)** | **P-value** |
| --- | --- | --- | --- | --- | --- | --- |
| **Right heart** |  |  |  |  |  |  |
| RVOT_vti_, m | 28/28 | 0.12 (0.01) | 0.13 (0.02) | 0.10 | -0.007 (-0.01;0.001) | 0.10 |
| TAPSE, mm | 29/26 | 18.1 (2.1) | 18.0 (2.3) | 0.77 | 0.2 (-1.0;1.4) | 0.74 |
| GLS, % | 22/19 | -26.4 (3.9) | -26.4 (3.9) | 0.99 | -0.2 (-2.7;2.2) | 0.86 |
| SV, ml | 25/24 | 25.1 (5.9) | 25.5 (5.9) | 0.79 | -0.4 (-3.2;2.3) | 0.77 |
| CO, L/min | 25/24 | 2.2 (0.7) | 2.1 (0.6) | 0.96 | 0.02 (-0.3;0.3) | 0.91 |
| TDI s’, cm/s | 29/25 | 9.3 (1.4) | 9.9 (1.5) | 0.11 | -0.6 (-1.4;0.2) | 0.13 |
| **Left heart** |  |  |  |  |  |  |
| LVOT_vti_, m | 28/24 | 0.15 (0.03) | 0.14 (0.02) | 0.39 | 0.006 (-0.008;0.02) | 0.40 |
| MAPSE, mm | 28/26 | 10.6 (1.2) | 11.7 (1.8) | 0.017 | -1.0 (-1.9;-0.2) | 0.019 |
| GLS, % | 22/19 | -21.3 (2.7) | -22.1 (3.9) | 0.48 | 0.7 (-1.5;2.9) | 0.51 |
| Shortening fraction, % | 29/28 | 0.33 (0.04) | 0.34 (0.05) | 0.39 | -0.01 (-0.03;0.01) | 0.40 |
| SV, ml | 27/24 | 20.9 (3.6) | 20.6 (3.4) | 0.74 | 0.4 (-1.5;2.2) | 0.70 |
| CO, L/min | 27/24 | 1.8 (0.4) | 1.7 (0.4) | 0.72 | 0.04 (-0.2;0.3) | 0.70 |
| **Septal** |  |  |  |  |  |  |
| TDI s’, cm/s | 29/27 | 5.9 (0.7) | 5.9 (0.7) | 0.85 | 0.05 (-0.3;0.4) | 0.79 |
| mpi’ | 29/26 | 0.37 (0.04) | 0.37 (0.05) | 0.69 | -0.004 (-0.03;0.02) | 0.74 |
| **Lateral** |  |  |  |  |  |  |
| TDI s’, cm/s | 29/25 | 5.5 (0.8) | 5.7 (1.1) | 0.29 | -0.3 (-0.8;0.3) | 0.32 |
| mpi’ | 28/25 | 0.38 (0.05) | 0.37 (0.04) | 0.91 | 0.002 (-0.03;0.03) | 0.89 |

Body surface area (BSA); confidence interval (CI); cardiac output (CO); global longitudinal strain (GLS) using 2-dimensional speckle tracking echocardiography; left ventricle outflow tract velocity time integral (LVOT_vti_); mitral annular plane systolic excursion (MAPSE); tissue Doppler imaging-derived myocardial performance index (mpi’); right ventricular outflow tract velocity time integral (RVOT_vti_); standard deviation (SD); stroke volume (SV); tricuspid annular plane systolic excursion (TAPSE); tissue Doppler imaging-derived annular systolic ejection velocity (TDI s’).

Data are shown as mean (SD).

Significant difference, P-value < 0.01.

† Crude value. ^a^ Accepted for analysis. ^b^ Mean difference adjusted for sex and BSA.

**Supplementary Table S7. Right and left heart diastolic function in 6.5-year-old children born extremely preterm (EPT), stratified by gestational age.**

|  | **Accepted^a^**  **GA 23-25 weeks**  **/GA26-27 weeks** | **Gestational age 23+0-25+6 weeks**  **n=29** | **Gestational age 26+0-27+6 weeks**  **n=28** | **P-value**† | **Adjusted mean difference^b^**  **(95% CI)** | **P-value** |
| --- | --- | --- | --- | --- | --- | --- |
| **Right heart** |  |  |  |  |  |  |
| TDI e’, cm/s | 29/25 | 13.5 (1.8) | 13.5 (2.0) | 0.96 | 0.03 (-1.0;1.1) | 0.96 |
| TDI a’ cm/s | 29/25 | 6.3 (2.0) | 6.1 (1.5) | 0.70 | 0.2 (-0.7;1.2) | 0.63 |
| e’/a’ | 29/25 | 2.3 (0.7) | 2.4 (0.7) | 0.86 | -0.05 (-0.4;0.3) | 0.79 |
| **Left heart** |  |  |  |  |  |  |
| **Septal** |  |  |  |  |  |  |
| TDI e’, cm/s | 29/27 | 11.4 (1.6) | 11.4 (1.1) | 0.91 | -0.04 (-0.8;0.7) | 0.91 |
| TDI a’, cm/s | 29/27 | 3.9 (0.9) | 3.9 (0.9) | 1.0 | -0.007 (-0.5;0.5) | 0.98 |
| e’/a’ | 29/27 | 3.1 (1.0) | 3.1 (0.8) | 0.86 | 0.04 (-0.5;0.5) | 0.88 |
| ivrt’, ms | 29/26 | 48.0 (7.1) | 47.2 (5.3) | 0.61 | 1.0 (-2.4;4.3) | 0.57 |
| ivct’, ms | 29/27 | 51.1 (6.4) | 52.0 (7.7) | 0.62 | -0.9 (-4.7;2.9) | 0.64 |
| ET, msec | 29/26 | 271 (17) | 269 (15) | 0.66 | 1.6 (-6.8;9.9) | 0.71 |
| **Lateral** |  |  |  |  |  |  |
| TDI e’, cm/s | 29/25 | 15.0 (2.7) | 16.0 (2.2) | 0.14 | -1.0 (-2.3;0.4) | 0.15 |
| TDI a’, cm/s | 29/25 | 4.2 (1.0) | 4.2 (1.1) | 0.93 | -0.02 (-0.6;0.6) | 0.94 |
| e’/a’ | 29/25 | 3.7 (0.9) | 4.0 (1.0) | 0.33 | -0.3 (-0.8;0.3) | 0.33 |
| ivrt’, ms | 28/25 | 50.1 (7.6) | 48.8 (6.9) | 0.54 | 1.1 (-2.9;5.1) | 0.59 |
| ivct’, ms | 28/25 | 55.5 (9.3) | 56.5 (9.2) | 0.69 | -0.8 (-6.0;4.3) | 0.75 |
| ET, msec | 28/25 | 282 (17) | 282 (19) | 1.0 | -0.1 (-10.5;10.3) | 0.98 |

Body surface area (BSA); confidence interval (CI); ejection time (ET); isovolumic contraction time (ivct’); isovolumic relaxation time (ivrt’); standard deviation (SD); tissue Doppler imaging-derived atrial (late) diastolic velocity (TDI a’); tissue Doppler imaging-derived early diastolic velocity (TDI e’).

Data are shown as mean (SD).

Significant difference, P-value < 0.01.

† Crude value. ^a^ Accepted for analysis. ^b^ Mean difference adjusted for sex and BSA.
